# Supplementary material for: Structure–Activity Relationship of Aloperine Derivatives as New Anti–Liver Fibrogenic Agents
Source: Molecules. 2020 Oct 27;25(21):4977. doi: 10.3390/molecules25214977 (PMC7663597; doi:10.3390/molecules25214977)
Supplement: Supplementary file 1 [file molecules-25-04977-s001.pdf]

Supporting Information

Structure–activity Relationship of Aloperine Derivatives as New Anti–Liver Fibrogenic Agents

Kun Wang, Zhihao Guo, Yunyang Bao, Yudong Pang, Yinghong Li*, Hongwei He*, Danqing Song

Institute of Medicinal Biotechnology, Chinese Academy of Medical Sciences and Peking Union Medical College, Beijing 100050, China; kunwang224@163.com (K. W.); guozhihao96@163.com (Z. G.) 17714333891@163.com (Y. B.); pyd101101@163.com (Y. P.); songdanqing@imb.pumc.edu.cn (D. S.).

* Correspondence: liyinghong@imb.pumc.edu.cn; Tel.:+86 10 63033012 (Y. L.) and hehwei@imb.pumc.edu.cn; Tel: +86 10 83166673 (H. H.)

Legends

Figure S1. The acute effects on luciferase activity of target compounds (**4h**, **4i**, **4n**, **4p** and **10g**)

Figure S2. The IC_{50} bar graphs of target compounds (**4h**, **4i**, **4n**, **4p** and **10g**)

Figure S3. The cell viability of target compounds in LX2 cells (**4h**, **4i**, **4n**, **4p** and **10g**)

Figure S4. The cell viability of target compounds in HepG2 cells (**4h**, **4i**, **4n**, **4p** and **10g**)

Table S1. P values of the RT-PCR of target compounds in Figure 2.

Table S2. P values of the western blot intensity of target compounds in Figure 3.

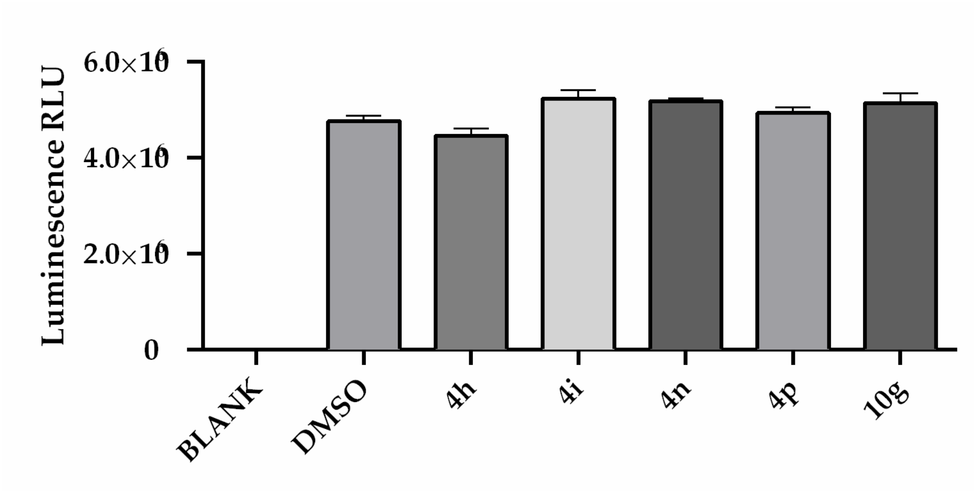

Figure S1. The acute effects on luciferase activity of target compounds (**4h**, **4i**, **4n**, **4p** and **10g**)

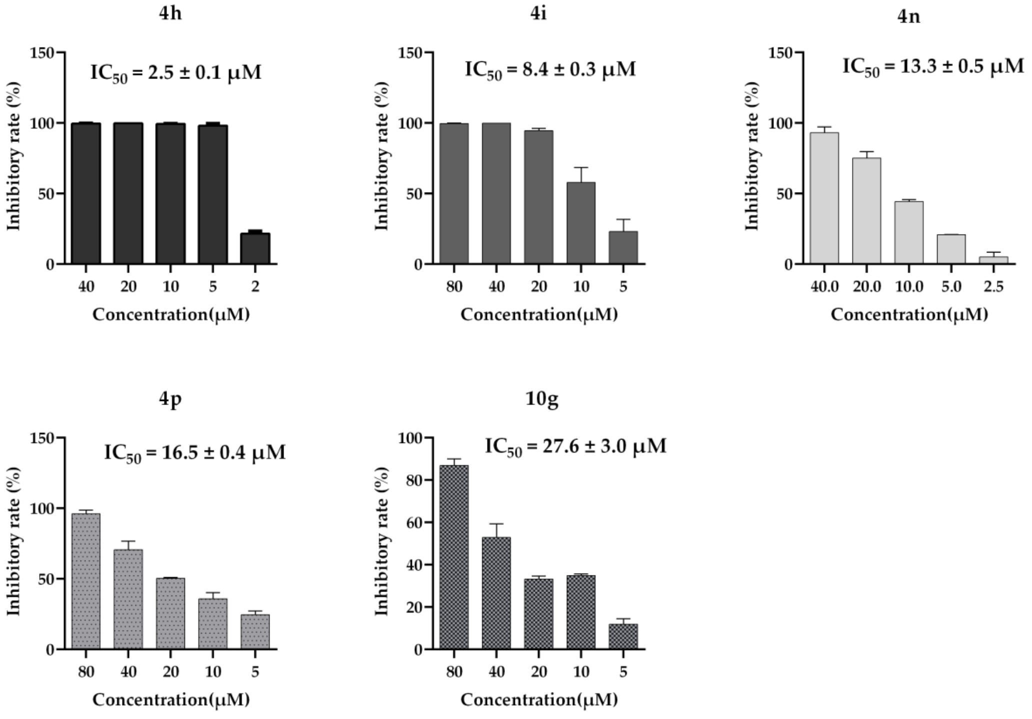

Figure S2. The IC_{50} bar graphs of target compounds (**4h**, **4i**, **4n**, **4p** and **10g**)

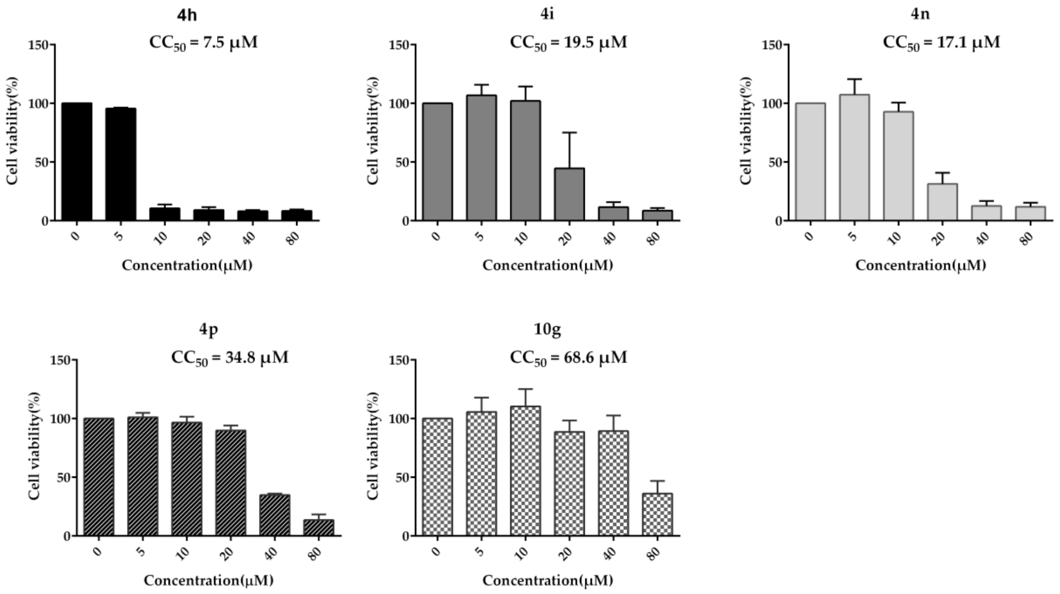

Figure S3. The cell viability of target compounds in LX2 cells (**4h**, **4i**, **4n**, **4p** and **10g**)

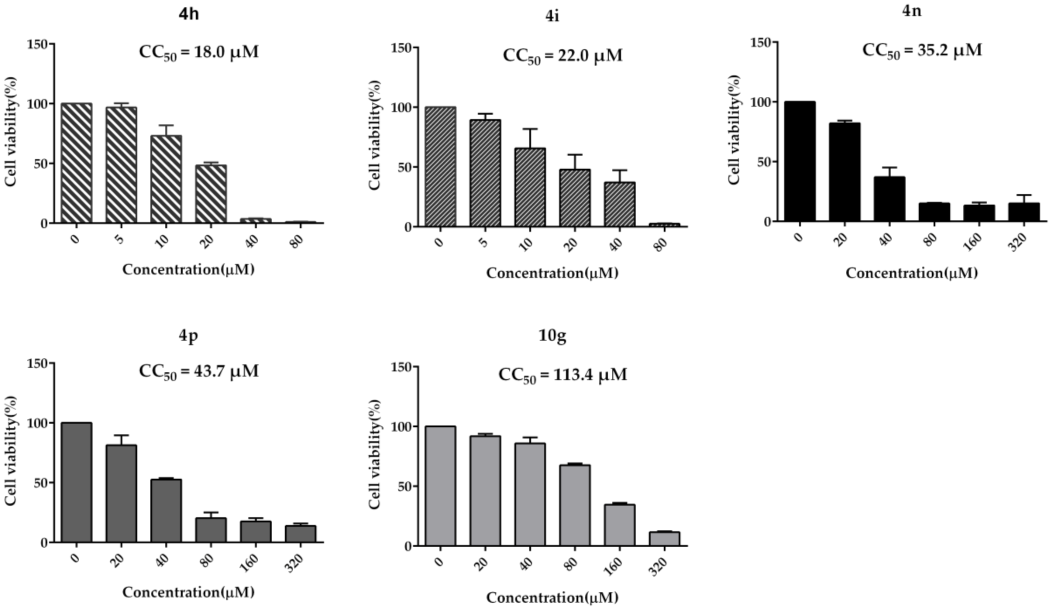

Figure S4. The cell viability of target compounds in HepG2 cells (**4h**, **4i**, **4n**, **4p** and **10g**)

Table S1. P values of the RT-PCR of target compounds.

Code	C (μM)	P value	
		COL1A1	TGF-β
TGF-β	2 ng/mL	0.01 [b]	0.01 [b]
**4n**+TGF-β[a]	6	0.003 [c]	0.03 [c]
	12	0.002 [c]	0.03 [c]
**4p**+TGF-β[a]	6	0.003 [c]	0.01 [c]
	12	0.001 [c]	0.03 [c]
**4i**+TGF-β[a]	12	0.004 [c]	0.02 [c]

[a] The concentration of TGF-β was 2 ng/mL; [b] compared to that of control group; [c] as compared to that of TGF-β1 group.

Table S2. P values of the western blot intensity of target compounds.

Code	C (μM) of compounds	P value		
		COL1A1	TGF-β	α-SMA
**4n**+TGF-β[a]	0	0.001 [b]	0.011 [b]	0.04 [b]
	6	0.202 [c]	0.177 [c]	0.02 [c]
	12	0.001 [c]	0.003 [c]	0.001 [c]
**4p**+TGF-β[a]	0	0.018 [b]	0.030 [b]	0.04 [b]
	6	0.162 [c]	0.433 [c]	0.02 [c]
	12	0.034 [c]	0.001 [c]	0.01 [c]

[a] The concentration of TGF-β was 2 ng/mL; [b] compared to that of control group; [c] as compared to that of TGF-β1 group.
